# Risk Factors for the Requirement of Antenatal Insulin Treatment in Gestational Diabetes Mellitus

**DOI:** 10.1155/2016/9648798

**Published:** 2016-11-23

**Authors:** Mayu Watanabe, Akihiro Katayama, Hidetoshi Kagawa, Daisuke Ogawa, Jun Wada

**Affiliations:** ^1^Department of Nephrology, Rheumatology, Endocrinology and Metabolism, Okayama University Graduate School of Medicine, Dentistry and Pharmaceutical Sciences, Okayama 700-8558, Japan; ^2^Department of Internal Medicine, Japanese Red Cross Society Himeji Hospital, Himeji, Hyogo 670-8540, Japan

## Abstract

Poor maternal glycemic control increases maternal and fetal risk for adverse outcomes, and strict management of gestational diabetes mellitus (GDM) is recommended to prevent neonatal and maternal complications. However, risk factors for the requirement of antenatal insulin treatment (AIT) are not well-investigated in the pregnant women with GDM. We enrolled 37 pregnant women with GDM and investigated the risk for AIT by comparing the patients with AIT (AIT group; *n* = 10) and without insulin therapy (Diet group; *n* = 27). The 1-h and 2-h plasma glucose levels and the number of abnormal values in 75 g OGTT were significantly higher in AIT group compared with Diet group. By logistic regression analysis, plasma glucose level at 1-h was significant predictor for AIT and the odds ratios were 1.115 (1.004–1.239) using forward selection method and 1.192 (1.006–1.413) using backward elimination method. There were no significant differences in obstetrical outcomes and neonatal complications. 1-h plasma glucose levels in 75 g OGTT are useful parameters in predicting the requirement for AIT in GDM. Both maternal and neonatal complications are comparable in GDM patients with and without insulin therapy.

## 1. Introduction

International Association of Diabetes and Pregnancy Study Groups (IADPSG) published criteria for the universal screening and diagnosis for gestational diabetes mellitus (GDM) based on Hyperglycemia and Adverse Pregnancy Outcome (HAPO) study [1]. After the release, the criteria for GDM were also updated in Japan [2] and they were characterized by two major revisions. First, GDM is diagnosed if one or more of the following criteria are met in the 75 g oral glucose tolerance test (OGTT) and threshold values were defined as a fasting plasma glucose level of 5.1 mmol/L (92 mg/dl), a 1-h plasma glucose level of 10.0 mmol/L (180 mg/dl), and a 2-h plasma glucose level of 8.5 mmol/L (153 mg/dl). Second, the new criteria exclude overt diabetes in pregnancy from GDM. By these new diagnostic criteria, the total incidence was 17.8% and FPG plus 1-h plasma glucose levels identified a large majority of these individuals [1].

Poor maternal glycemic control can significantly increase maternal and fetal risk for adverse outcomes [3] and strict management of GDM is recommended to prevent neonatal and maternal complications. Several studies have shown the treatment interventions, including dietary treatment, self-monitoring blood glucose levels, and insulin therapy if required, reduced prenatal complications [4, 5]. Crowther et al. found that 20 percent of women with GDM need antenatal insulin treatment (AIT) to achieve good glycemic control [6], while other studies also showed that 10.8~52.8 percent of GDM patients required AIT [7–11]. In addition, Baptiste-Roberts et al. reviewed that the AIT was the risk factor for the development of type 2 diabetes [12]. However, the risk factors for the requirement of AIT are not well-investigated and this prompted us to investigate the clinical characteristics of the patients with GDM to identify risk factors for AIT.

## 2. Materials and Methods

### 2.1. Subjects

The current clinical investigation is a noninterventional and retrospective study enrolling 37 pregnant women with GDM. They were diagnosed at 35.2 ± 4.7 years of age at their estimated 20.9 ± 3.8 gestational weeks and admitted between 2010 and 2016 at Himeji Red Cross Hospital, Hyogo, Japan. GDM was diagnosed according to IADPSG criteria [1]. Data collected included gestational age at diagnosis, gestational weeks at diagnosis, twins, family history of diabetes mellitus, pregestational body mass index (BMI), infertility treatments such as timed intercourse,* in vitro* fertilization, and egg donation, hospitalization for threatened premature delivery and intravenous ritodrine hydrochloride, prior gestational diabetes, primipara, plasma glucose levels of 75 g OGTT (fasting, 1-h, and 2-h), number of abnormal values of 75 g OGTT, glycosylated hemoglobin A1c (HbA1c), weeks of gestation at start of insulin therapy, the maximum of insulin dose, obstetrical outcome such as weeks of gestation at delivery, preterm delivery, cesarean section, pregnancy-induced hypertension (PIH), and neonatal characteristics such as birth weight, neonatal plasma glucose levels, hypoglycemia defined as plasma glucose levels of less than 45 mg/dL, transient tachypnea of the newborn (TTN), and respiratory distress syndrome (RDS). We excluded the patients with overt diabetes in pregnancy, with known type 1 or type 2 diabetes before pregnancy, or having not undergone 75 g OGTT. Furthermore, pregnant women were classified as normal weight (BMI < 25 kg/m^2^), overweight (25 ≤ BMI < 30 kg/m^2^), and obese (30 ≤ BMI kg/m^2^) according to pregestational BMI. The study protocol was approved by the institutional review boards of Himeji Red Cross Hospital.

### 2.2. Treatments

The patients received dietary education from registered dietitians with 30 kcal/kg weight of ideal body weight based on BMI 22 kg/m^2^ supplemented with 200 to 250 kcal, and they were instructed to take three meals and one to three snacks. They also received instructions for the procedures to perform self-monitoring of blood glucose (SMBG) and measured daily both preprandial and 2-hour postprandial glucose levels. If targeted glucose levels with preprandial glucose less than 5.6 mmol/L (100 mg/dl) and 2-hour postprandial glucose less than 6.7 mmol/L (120 mg/dl) were not achieved three times or more after breakfast, lunch, and dinner during seven days, AIT was initiated before breakfast, lunch, and dinner, respectively. We classified the patients into two groups, the patients who received AIT (AIT group; *n* = 10) and the patients without insulin therapy (Diet group; *n* = 27).

### 2.3. Statistical Analysis

All data were presented as the mean ± standard deviation. For univariate analysis, we used Student's* t*-test and *χ*-square test for categorical data. For multivariate logistic regression analysis after controlling simultaneously for potential confounders, we selected independent variables which were significantly higher in AIT group compared with Diet group for univariate analysis, such as postprandial PG at 1-hr and 2-hr in 75 g OGTT and number of abnormal values in 75 g OGTT. We also used the forward selection and backward elimination methods. Logistic regression analyses with each independent variable to explore risk factors contributing to AIT were also performed. We performed receiver-operating characteristic (ROC) curve to identify clinical factors to predict the requirement for AIT. We determined a cut-off value by the point on the ROC curve closest to the upper left corner. *P* values of less than 0.05 were considered statistically significant. The data were analyzed with IBM SPSS Statistics Ver. 22.0 and IBM SPSS Regression (IBM).

## 3. Results

### 3.1. Characteristics of GDM

Maternal age, number of twins, family history of DM, pregestational BMI, infertility treatment, prior gestational diabetes, primipara, fasting plasma glucose levels at 75 g OGTT, and HbA1c levels demonstrated no significant differences between AIT and Diet groups. The gestational age at diagnosis was lower in AIT group (18.9 ± 2.0 weeks) compared with Diet group (21.6 ± 4.1 weeks), but it did not reveal significant differences (*P* = 0.053). The 1-h and 2-h plasma glucose levels and number of abnormal values in 75 g OGTT were significantly higher in AIT group compared with Diet group (Table 1). The maximal glucose level during ritodrine treatment in AIT group was higher compared to Diet group, the intravenous injection of ritodrine to treat preterm delivery was more frequently administered in AIT group (40.0%) compared with Diet group (14.8%), but it was not statistically significant (Table 1).

### 3.2. Risk Factors for AIT

**Figure 1:**
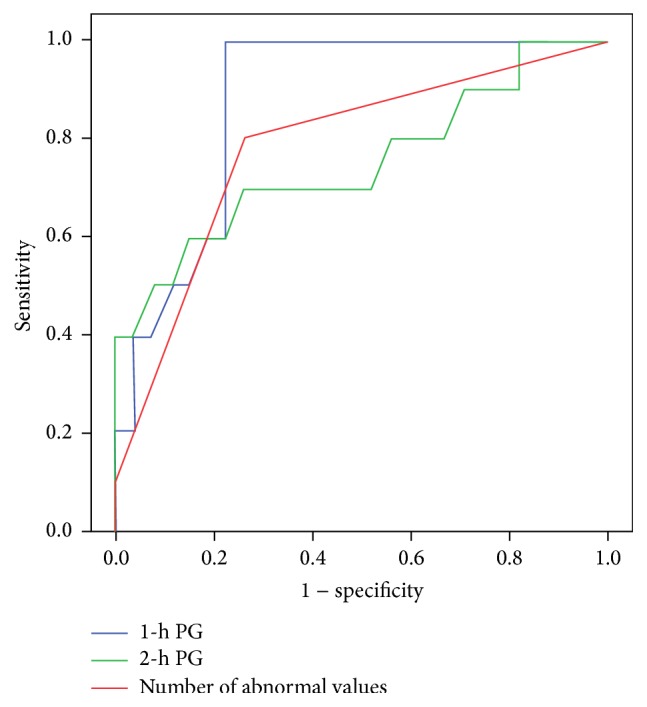


By logistic regression analysis, plasma glucose level at 1-h was significant predictor for AIT and the odds ratios were 1.115 (1.004–1.239) using forward selection method and 1.192 (1.006–1.413) using backward elimination method (Table 2). In logistic analyses using each independent variable, plasma glucose levels at 1-h and 2-h and the number of abnormal values in 75 g OGTT were only significant predictors for AIT and the odds ratios were 1.128 (1.022–1.246), 1.054 (1.006–1.104), and 10.950 (1.959–61.218), respectively (Table 3). ROC curves were used to determine the cut-off values of 1-h and 2-h plasma glucose levels and the number of abnormal values to predict the AIT (Figure 1). The cut-off values of 1-h and 2-hr plasma glucose levels and the number of abnormal values in 75 g OGTT were 10.25 mmol/L (AUC 0.872, sensitivity 100%, and specificity of 77.8%), 8.75 mmol/L (AUC 0.756, sensitivity 70%, specificity 70.4%), and 1.5 (AUC 0.783, sensitivity 80%, and specificity 74.1%), respectively (Table 4).

### 3.3. Obstetrical Outcomes and Neonatal Characteristics

The rate of cesarean section and PIH was higher in AIT group compared with Diet group, but it was not statistically significant (Table 5). There were no differences in the rate of preterm delivery; however, gestational age at delivery was significantly lower in AIT group (36.4 ± 2.0 weeks) compared with Diet group (38.2 ± 2.4 weeks) (Table 5). There was no stillbirth, Apgar score at 5 minutes less than 8, shoulder dystopia, and birth weight more than 4000 grams. TTN occurred more often in AIT group compared with Diet group without significant differences (Table 6). There was no difference in the rate of RDS between two groups. Birth weight, neonatal plasma glucose levels, and the rate of hypoglycemia were similar in both groups (Table 6).

## 4. Discussion

The main finding in the current investigation is that 1-h and 2-h plasma glucose levels and the number of abnormal values in 75 g OGTT predict the requirement for AIT in GDM patients. The number of abnormal OGTT values was 1.5; therefore two or three of abnormal values in 75 g OGTT are a predictor for need of AIT with sensitivity of 90% and specificity 35.5%. Ikenoue et al. evaluated the insulin sensitivity, insulin secretion, and *β* cell function with insulin secretion-sensitivity index-2 (ISSI-2) in women between one and two or three abnormal OGTT values. The insulin sensitivity of women with two or three abnormal values deteriorated significantly compared with one abnormal value, although the insulin secretions were similar between two groups. Consequently, ISSI-2 levels of women with two or three abnormal values were significantly lower compared to one abnormal value. They also found that women with two or three abnormal OGTT values required more frequently AIT compared to one abnormal value [13]. Saisho et al. found that Japanese women with GDM failed to increase insulin secretion to compensate decreased insulin sensitivity compared to women with normal glucose tolerance [14]. Therefore, the number of abnormal OGTT values might reflect insulin sensitivity and *β* cell function of women with GDM.

Previous studies reported diverse possible factors predicting the need for AIT including HbA1c, plasma glucose levels in OGTT, pregestational BMI, maternal age, gestational age at diagnosis, and family history of diabetes [7–11], but they were not associated with the requirement for AIT in the current study. The differences in risk factors among the previous and current studies may be derived from the clinical characteristics of the participants. For example, several studies showed that fasting glucose level could be predictor for AIT among women with GDM [7, 9–11, 15]. The mean of pregestational BMI among women who required AIT was reported as 31.6 kg/m^2^ [11] and 29.9 kg/m^2^ [7]. The proportion of overweight and obese women in AIT group was 72.9 percent [7]. In the current study, the mean of pregestational BMI among women who required AIT was 24.5 kg/m^2^. The proportion of overweight and obese women in AIT group was 40 percent; the current study included overweight and obese woman, but prevalence was less compared to the previous studies. Black et al. found the association between BMI and fasting glucose levels, and obese women with GDM had significantly higher mean fasting glucose levels compared to overweight and normal weight women with GDM [16]. In the current study, indeed fasting glucose levels were significantly different between overweight or obese and normal weight women in Diet group, but fasting glucose levels were not significantly different between overweight, obese women and normal weight women in AIT group (Table 7). In the current study, the proportion of overweight and obese women of participants was less and fasting glucose level was not important risk factor for AIT.

*β*-Adrenergic agonist (ritodrine) infusion has been used as tocolytic; however it has some maternal side effects such as hyperglycemia. Even in nondiabetic pregnancy in women with normal glucose tolerance, it raised blood glucose levels additional 2.2 mmol/L (40 mg/dl) [17] and the continuous ritodrine infusion increased plasma glucose levels during first 24 hours after beginning of ritodrine [18]. In contrast, the Canadian preterm labor investigators group reported that the incidences of hyperglycemia were similar between ritodrine and placebo infusion group [19]. In the current study there were four women to treat preterm labor with ritodrine in each group (Table 1), and there was one woman with twin in Diet group, as well as two women with twin in AIT group. Twin was risk factor of preterm labor [20]. In the current study, all three women with twin had treatment of preterm labor with ritodrine (Table 1). That was one of the reasons that AIT group had higher frequency of ritodrine, although there were no significant differences in the number of the women given ritodrine to arrest preterm delivery between AIT and Diet group.

There are some limitations in the current study. First, the number of participants was small to assess the predictors of requirement for AIT and it underestimated other important factors. Second, current study was single-center retrospective investigation. The prospective cohort study in larger participants is needed to further determine risk factors to predict AIT.

In conclusion, 1-h and 2-h plasma glucose levels and number of abnormal values in 75 g OGTT are useful parameters in predicting the requirement for AIT in GDM. Both maternal and neonatal complications are comparable in GDM patients with and without insulin therapy.

## Figures and Tables

**Table 1:** Characteristics of GDM.

	AIT group (*n* = 10)	Diet group (*n* = 27)	*P* value
Gestational age at diagnosis (years)	33.8 ± 7.0	35.7 ± 3.6	0.284^†^
Gestational weeks at diagnosis (weeks)	18.9 ± 2.0	21.6 ± 4.1	0.053^†^
Twins, *n* (%)	2 (20.0)	1 (3.7)	0.107^‡^
Family history of diabetes mellitus, *n* (%)	7 (70.0)	16 (59.3)	0.550^‡^
Pregestational BMI (kg/m^2^)	24.5 ± 4.8	23.0 ± 4.4	0.383^†^
95% CI	21.0–27.9	21.3–24.7	
<20.0 (%)	20.0	14.8	
20–24.9 (%)	40.0	59.3	
25–29.9 (%)	30.0	18.5	
≥30.0 (%)	10.0	7.4	
Infertility treatment, *n* (%)	4 (40.0)	12 (44.4)	0.809^‡^
Treatment of preterm labor with ritodrine, *n* (%)	4 (40.0)	4 (14.8)	0.098^‡^
Maximal glucose levels during ritodrine treatment (mmol/L)	9.0 ± 1.2	7.3 ± 0.7	0.062^†^
Prior gestational diabetes, *n* (%)	1 (10.0)	5 (18.5)	0.532^‡^
Primipara, *n* (%)	5 (50.0)	12 (44.4)	0.763^‡^
Plasma glucose level (mmol/L)			
Fasting^¶^	4.82 ± 0.49	4.69 ± 0.46	0.465^†^
At 1 hr^§^	10.86 ± 0.62	9.19 ± 1.57	0.003^†^
At 2 hr^§^	9.43 ± 1.56	7.96 ± 1.37	0.008^†^
Number of abnormal values, *n*	1.9 ± 0.6	1.26 ± 0.4	0.001^†^
HbA1c (%)	5.3 ± 0.3	5.2 ± 0.4	0.234^†^
Gestational weeks at start of AIT (weeks)	26.1 ± 5.9		
The maximum of insulin dose (units/day)	10 ± 5.5		

AIT, antenatal insulin treatment; BMI, body mass index; CI, confidence interval; and HbA1c, hemoglobin A1c.

Data are shown as mean ± SD or *n*.

^†^By Student's *t*-test.

^‡^By Chi-square test.

^¶^At the time of the 75 g oral glucose tolerance test.

^§^After the administration of 75 g of glucose.

**Table 2:** Logistic regression analysis after controlling simultaneously for potential confounders of predictors for antenatal insulin treatment (AIT).

Independent variables	*β*-Coefficient	Odds ratio (95% CI)	*P* values
Postprandial PG at 1-hr in 75 g OGTT	0.165	1.179 (0.988–1.407)	0.067
Postprandial PG at 2-hr in 75 g OGTT	0.053	1.054 (0.987–1.126)	0.113
Number of abnormal values in 75 g OGTT	0.420	1.521 (0.207–11.201)	0.680

*Forward selection method (conditional)*			
Postprandial PG at 1-hr in 75 g OGTT	0.109	1.115 (1.004–1.239)	0.043
Number of abnormal values in 75 g OGTT	1.451	4.267 (0.669–27.228)	0.125

*Backward elimination method (conditional)*			
Postprandial PG at 1-hr in 75 g OGTT	0.176	1.192 (1.006–1.413)	0.042
Postprandial PG at 2-hr in 75 g OGTT	0.059	1.061 (0.998–1.127)	0.057

AIT, antenatal insulin treatment; PG, plasma glucose; 75 g OGTT, 75 g oral glucose tolerance test, BMI, body mass index, and HbA1c, hemoglobin A1c.

**Table 3:** Logistic regression analysis with each independent variable of predictors for antenatal insulin treatment (AIT).

	*β*-Coefficient	Odds ratio (95% CI)	*P* values
Postprandial PG at 1-hr in 75 g OGTT	0.121	1.128 (1.022–1.246)	0.017
Postprandial PG at 2-hr in 75 g OGTT	0.053	1.054 (1.006–1.104)	0.026
Number of abnormal values in 75 g OGTT	2.393	10.950 (1.959–61.218)	0.006

AIT, antenatal insulin treatment; PG, plasma glucose; and 75 g OGTT, 75 g oral glucose tolerance test.

**Table 4:** The cut-off values of 1-h and 2-h plasma glucose levels and the number of abnormal values to predict antenatal insulin treatment (AIT).

	Cut-off point	Sensitivity	Specificity	AUC
Postprandial PG at 1-hr in 75 g OGTT	10.25 mmol/L	100%	77.8%	0.872
Postprandial PG at 2-hr in 75 g OGTT	8.75 mmol/L	70%	70.4%	0.756
Number of abnormal values in 75 g OGTT	1.5	80%	74.1%	0.783

AUC, area under the curve; PG, plasma glucose; and 75 g OGTT, 75 g oral glucose tolerance test.

**Table 5:** Obstetrical outcomes.

	AIT group (*n* = 10)	Diet group (*n* = 27)	*P* values
Gestational age at delivery (week)	36.4 ± 2.0	38.2 ± 2.4	0.046^†^
Maternal weight gain (kg)	6.24 ± 5.46	7.27 ± 3.93	0.531^†^
Preterm delivery, *n* (%)	3 (30.0)	5 (18.5)	0.451^‡^
Cesarean section, *n* (%)	7 (70.0)	10 (37.0)	0.074^‡^
PIH, *n* (%)	3 (30.0)	2 (7.4)	0.074^‡^

Data are shown as mean ± standard deviation (SD) or number (*n*). AIT, antenatal insulin; PIH, pregnancy-induced hypertension; ^†^Student's *t*-test; and ^‡^ *χ*-square test.

**Table 6:** Neonatal characteristics.

	AIT group (*n* = 10)	Diet group (*n* = 27)	*P* values
Birth weight (g)	2703.2 ± 526.0	2827.6 ± 534.3	0.532^†^
Neonatal plasma glucose level (mmol/L)	2.3 ± 1.3	3.0 ± 1.0	0.099^†^
Hypoglycemia, *n* (%)	4 (40)	5 (18.5)	0.176^‡^
TTN, *n* (%)	3 (30.0)	2 (7.4)	0.074^‡^
RDS, *n* (%)	2 (20.0)	1 (3.7)	0.107^‡^

Data are shown as mean ± standard deviation (SD) or number (*n*). AIT, antenatal insulin; TTN, transient tachypnea of the newborn; RDS, respiratory distress syndrome; ^†^Student's *t*-test; and ^‡^ *χ*-square test.

**Table 7:** 

	AIT group (*n* = 10)		
	Pregestational BMI < 25.0 (kg/m^2^)(*n* = 6)	Pregestational BMI ≥ 25.0 (kg/m^2^) (*n* = 4)	*P* value
Fasting plasma glucose level (mmol/L) ^¶^	4.6 ± 0.4	5.2 ± 0.5	0.071^†^

	Diet group (*n* = 27)		
	Pregestational BMI < 25.0 (kg/m^2^) (*n* = 20)	Pregestational BMI ≥ 25.0 (kg/m^2^) (*n* = 7)	*P* value

Fasting plasma glucose level (mmol/L) ^¶^	4.6 ± 0.4	5.0 ± 0.4	0.031^†^

AIT, antenatal insulin treatment; BMI, body mass index.

Data are shown as mean ± SD.

^†^By Student's *t*-test.

^¶^At the time of the 75 g oral glucose-tolerance test.
